# Prevalence of cytomegalovirus infection in patients with ulcerative colitis: a prospective cross-sectional study in Tehran, Iran

**Published:** 2018-10

**Authors:** Davood Yadegarynia, Shabnam Tehrani, Manijeh Roohi, Latif Gachkar, Seyed Alireza Nadji, Mohammad Hashemi, Saadat Molanaei

**Affiliations:** 1 Infectious Diseases and Tropical Medicine Research Center, Shahid Beheshti University of Medical Sciences, Tehran, Iran; 2 Virology Research Center, National Research Institute of Tuberculosis and Lung Diseases, Shahid Beheshti University of Medical Sciences, Tehran, Iran; 3 Imam Hossein Hospital, Shahid Beheshti University of Medical Sciences, Tehran, Iran; 4 Department of Pathology, Milad Hospital, Tehran, Iran

**Keywords:** Ulcerative colitis, Cytomegalovirus, Real-time polymerase chain reaction, Iran

## Abstract

**Background and Objectives:**

Cytomegalovirus (CMV) infection has been reported in ulcerative colitis (UC), but limited data are available on its prevalence in Iran. The aim of this study was to evaluate the prevalence of CMV infection in patients with UC.

**Materials and Methods:**

A prospective, cross-sectional study was conducted in 86 consecutive patients with UC. Prevalence of CMV infection was determined by rectal biopsies for hematoxylin and eosin staining and PCR. CMV-positive specimens was measured for CMV loads by real-time PCR assay.

**Results:**

In six out of 86 (7%) patients with UC, CMV was diagnosed. These patients had detectable CMV DNA in their biopsies as indicated by PCR. In all CMV-positive patients, viral load was more than 250 copy/mg. Histochemical staining did not show any CMV inclusion bodies. No significant demographic and clinical differences existed between patients with and without a CMV infection.

**Conclusion:**

UC and its treatment may put patients at risk of CMV infection. Real-time PCR test for the detection of CMV in UC patients may enable diagnosis of CMV infection with a high sensitivity and allow effective treatment to be administered in these patients. The impact of antiviral therapy on the clinical outcome of the UC patients with CMV remains to be elucidated.

## INTRODUCTION

Cytomegalovirus (CMV) belongs to the herpesvirus family, and is quite prevalent in adults (1). From 40 to 100% of adults possesses an antibody to CMV (2, 3). The virus usually cause severe disease in immunocompromised patients, such as those with inflammatory bowel disease (IBD) as these patients receive multiple immunosuppressive drugs (4–7). Therefore, patients with IBD (i.e. ulcerative colitis (UC) and Crohn’s disease (CD)) are expected to be at an increased risk of CMV infection (8–10). Although, some authors from different parts of the world have reported the prevalence of CMV infection in patients with IBD, limited data are available in Iran (11–13). Furthermore, only a few studies used PCR for diagnosis of CMV infection in IBD (8). Unrecognized CMV infection in IBD patients may cause fulminant disease, requiring colectomy and even death (3). Thus, having a reliable estimate of the disease as well as early diagnosis of CMV can significantly decrease the morbidity and mortality. The present study was designed to estimate the prevalence of CMV infection in patients with UC in Tehran, Iran and to compare pathological results with PCR for detection of this infection.

## MATERIALS AND METHODS

### Patients.

This cross-sectional prospective study was conducted from March to December 2017 in two hospitals of Tehran, Khatamolanbia and Milad. Eighty-six consecutive patients who had been diagnosed with UC were randomly included in the study. All patients signed the consent form and agreed to participate in the study. A questionnaire form was created about patient’s age, gender, occupation, education, duration of illness, ethnicity, medications, interventions, family history, location of colon involvement and steroid therapy in the last week, which was filled by calling each patient. The diagnosis of UC had been established by clinical, colonoscopic and histologic findings. Ethics committee of Shahid Beheshti University of Medical Sciences approved the study protocol.

### Collection of samples.

Biopsies were obtained during colonoscopy for histological examination of CMV inclusion bodies and inflammatory cells, and for extraction of DNA for PCR. Colonoscopic biopsies collected in normal saline were stored at −80°C until further processing for CMV DNA.

### Histopathology.

Colonic biopsies were paraffinized, sectioned and stained with haematoxylin and eosin. These sections were evaluated under a microscope for characteristics cytomegalic cells and ‘owl’s-eye’ nuclear inclusion bodies. Histologically, heavy inflammatory infiltrate with epithelial ulceration considered as active disease (8).

### Nucleic acid extraction from tissues.

Extraction of total DNA from the tissue samples was performed using the RTP® DNA/RNA virus mini kit procedure (Stratec molecular, Berlin, Germany). The extracted nucleic acid was stored at −20°C until it under went PCR.

### PCR and Real-Time PCR.

To assess the quality of the extracted genome, as well as inhibition of the PCR test, all extracted and stored nucleic acid underwent beta-globin PCR, using the PCO3/PCO4 primer set as described previously with modifications in the method (14). The β-glubin PCR was performed in SYBER green real-time PCR-melting curve format.

To detect CMV, PCR was performed using envelope glycoprotein B (gpUL55) gene primer sets amplifying 116 bp gene region of the virus genome CMV-r; 5′-AAGTACCCCTATCGCGTGTG-3′, CMV-f; 5′-ATGATGCCCTCRTCCARGTC-3′, with an internal probe, CMV-P; 5′-FAM-TGGCCCAGGGTACGGATCTTATTCG-BHQ1-3′ (15). Amplification of CMV gpUL55 gene was performed in reaction volumes of 20 μL under the following conditions; first the samples underwent denaturation at 94°C for 10 minutes, followed by denaturation at 94°C for 10 seconds, followed by annealing and extension at 60°C for one minute, 50 cycles. CFX96 real-time PCR system (Bio-Rad, USA) with HS prime taq premix TaqMan reagent (GENETBIO, Korea) was used for quantitative analysis. The limit detection of five genome copies of CMV per reaction was determined by the real-time assay, using the serial dilutions of AmpliRun® CYTOMEGALOVIRUS DNA CONTROL (Vircell, Spain). In positive cases, the CMV viral load was determined using RealStar® CMV PCR Kit 1.0 (altona Diagnostics GmbH, Germany) in related samples.

Patients with viral load or quantitative CMV PCR more than 250 copy/mg, were considered as CMV-positive patients (16–18).

## RESULTS

Of 86 UC patients, 40 were male and 46 were female with an age range 15–80 years. The clinical and demographic characteristics of patients with and without CMV are shown in Table 1. The patients were different in disease characteristics such as duration of the UC, treatment regimens, and interventions. Duration of disease varied from less than one month to greater than thirty years.

**Table 1.** Demographic and clinical parameters of patients with UC with or without CMV infection

**Variables**	**CMV Negative**	**CMV Positive**	**Significant levels of difference**
Gender			P Value

Women	44 (95.7%)	2 (4.3%)	>0.05
Men	36 (90%)	4 (10%)	

Age (Years)			P Value

10–20	6 (100%)	0	
20–30	7 (77.8%)	2 (22.2%)	
30–40	17 (94.4%)	1 (5.6%)	>0.05
40–50	23 (100%)	0	
50<	27 (90%)	3 (10%)	

Duration of disease (Years)			P Value

<1	35 (94.6%)	2 (5.4%)	
1–5	19 (90.5%)	2 (9.5%)	>0.05
5–10	10 (90.9%)	1 (9.1%)	
10<	16 (94.1%)	1 (5.9%)	

Use of steroid			P Value

Yes	40 (90.9%)	4 (9.1%)	
No	38 (95%)	2 (5%)	>0.05
Not remember	2 (100%)	0	

Use of azaram			P Value

Yes	24 (88.9%)	3 (11.1%)	>0.05
No	56 (94.9%)	3 (5.1%)	

Drugs received			P Value

None	22 (27.5%)	0	
Sulfa or Meza	42 (52.5%)	3 (50%)	
Sulfa+Steroid	6 (7.5%)	0	
Sulfa+Azaram+Steroid	5 (6.25%)	2 (33.3%)	>0.05
Sulfa+Azaram	5 (6.25%)	0	
Meza+Celcept	0	1 (16.6%)	

Flare up			P Value

No	51 (96.2%)	2 (3.8%)	
1 per year	12 (92.3%)	1 (7.7%)	
2 per year	6 (85.7%)	1 (14.3%)	>0.05
3<year	2 (100%)	0	
No respond	5 (100%)	0	
Colectomy	6 (75%)	(225%)	

Site of involvement			P Value

Colon	43 (53.7%)	4 (66.6%)	
Pancolit	1 (1.25%)	1 (16.6%)	
Rectum	20 (25%)	1 (16.6%)	>0.05
Colonrectum	5 (6.25%)	0	
Rectusigmoid	11 (13.7%)	0	

Family history	Neg	POS	P Value

Yes	7 (8.25%)	1 (16.6%)	
No	73 (91.25%)	5 (83.3%)	>0.05

Of 86 patients, 6 (7.0%) had evidence of CMV infection. These patients had detectable CMV DNA in their biopsies as indicated by PCR. In all CMV-positive patients, viral load was more than 250 copy/mg. However, histochemical staining did not show any CMV inclusion bodies in UC patients. Of 6 CMV-positive patients, four were male and two were female with age range 26–80 years and the average of 46.8 years. Among the patients, 17 were<30 years of whom two were CMV positive and 69 patients were ≥30 years, where four were among CMV-positive patients. In CMV-positive cases, 67% of patients had immunosuppressive therapies and steroids in their treatment regimen. However, immunosuppressive therapies was not significantly associated with CMV infection (P>0.05). Among patients with positive CMV, one showed acute fulminant hemorrhagic colitis and died. Colectomy was done for 2 (30%) of 6 CMV-positive patients and 6 (7.5%) of 80 CMV-negative patients. Two patients who underwent colectomy were treated with cyclosporine, steroid pulse, and azathioprine before surgery. However, patients did not respond to treatment, and then pan-colectomy was performed. Demographic and clinical parameters were compared in patients with and without a CMV infection; however, no significant differences were found (Table 1).

## DISCUSSION

In the current study, the prevalence of CMV in patients with UC was 7%. This rate of CMV was lower than those of previous studies investigated the prevalence of CMV in IBD cases. During 2007–2008, Kim and others reported that 43% of patients with UC were infected with CMV (19). Likewise, subsequent investigation observed nearly the same percentage (3, 11, 20, 21). These studies supports the hypothesis that UC is highly associated with CMV infections (19). Though, the role of CMV in UC has been controversial. Early studies have highlighted the association of CMV infection with IBD and treatment with steroids (12, 20). while, others have indicated a lack of correlation of CMV and clinical severity of IBD (22). In our study, 44 patients with UC have received systemic corticosteroids; however, the use of corticosteroids was not significantly associated with CMV infection.

In our study, CMV infection was not significantly related to clinical parameters, and these results were consistent with earlier reports (11, 20).

In the present study, inclusion bodies were not seen in the specimens of included patients, but PCR as well as real-time PCR for CMV DNA were positive in six cases. In general, histochemical staining and molecular methods are recommended for diagnosis of CMV disease (23–25). Similar to our results, inclusion bodies sometimes may not be seen in specimens (26). Thus, molecular methods may have higher sensitivity and specificity when compared with pathological findings. Consistent with our findings, PCR of CMV DNA from colonic tissue exhibits high sensitivity (92% to 96.7%) and specificity (93% to 98.7%) (27, 28). Consequently, PCR may be helpful for diagnosis of CMV infection in suspected cases with negative histochemical staining. McCurdy et al. showed that CMV disease was significantly associated with age >30 years (29). Although in our study, age was not significantly associated with CMV infection, but among six CMV-positive patients, four (66.6%) were ≥ 30 years.

Several studies showed that UC patients with CMV infection had higher colectomy procedures than those without it (3, 30, 31). Domenech et al. reported colectomy in 3 (50%) of 6 CMV-positive patients and 2 (16.6%) of 12 CMV-negative patients (P=.054) (20). Likewise, in our study 2 (33.3%) of 6 CMV-positive patients and 6 (7.5%) of 80 CMV-negative patients underwent colectomy. Two CMV-positive patients who underwent colectomy had used steroid pulse, azathioprine and cyclosporine but their disease was not controlled and then pan-colectomy was performed. In CMV-positive group, 67% of patients had immunosuppressive therapies and steroids in their treatment regimen whiles in CMV-negative group it was 50%. Fukuchi et al. reported that 29.4% of UC patients without any previous immunosuppressive therapy had CMV DNA in their colon biopsies (32).

In conclusions, UC and its treatment may put patients at risk of CMV infection. Real-time PCR test for the detection of CMV in UC patients may enable diagnosis of CMV infection with a high sensitivity and allow effective treatment to be administered in these patients. The impact of antiviral therapy on the clinical outcome of the UC patients with CMV remains to be elucidated.
